# Extreme temperature combined with hypoxia, affects swimming performance in brown trout (*Salmo trutta*)

**DOI:** 10.1093/conphys/coz108

**Published:** 2020-01-23

**Authors:** Robert L Nudds, Karlina Ozolina, Miriam Fenkes, Oliver H Wearing, Holly A Shiels

**Affiliations:** Faculty of Biology, Medicine and Health, School of Biological Sciences, University of Manchester, Oxford Road, Manchester M13 9PL, UK

**Keywords:** Climate change, energetics, fish, kinematics, locomotion, physiology

## Abstract

Climate change is predicted to impact freshwater aquatic environments through changes to water temperature (*T*_water_), river flow and eutrophication. Riverine habitats contain many economically and ecologically important fishes. One such group is the migratory salmonids, which are sensitive to warm *T*_water_ and low O_2_ (hypoxia). While several studies have investigated the independent effects of *T*_water_ and hypoxia on fish physiology, the combined effects of these stressors is less well known. Furthermore, no study has investigated the effects of *T*_water_ and O_2_ saturation levels within the range currently experienced by a salmonid species. Thus, the aim of this study was to investigate the simultaneous effects of *T*_water_ and O_2_ saturation level on the energetics and kinematics of steady-state swimming in brown trout, *Salmo trutta*. No effect of O_2_ saturation level (70 and 100% air saturation) on tail-beat kinematics was detected. Conversely, *T*_water_ (10, 14, 18 and 22°C) did affect tail-beat kinematics, but a trade-off between frequency (*f*_tail_) and amplitude (*A*, maximum tail excursion) maintained the Strouhal number (St = *f*_tail_• *A*/*U*, where *U* is swimming speed) within the theoretically most mechanically efficient range. Swimming oxygen consumption rate (${\dot{M}}_{{\mathsf{O}}_{\mathsf{2}}}$) and cost of transport increased with both *U* and *T*_water_. The only effect of O_2_ saturation level was observed at the highest *T*_water_ (22°C) and fastest swimming speed (two speeds were used—0.6 and 0.8 m s^−1^). As the extremes of this study are consistent with current summer conditions in parts of UK waterways, our findings may indicate that *S. trutta* will be negatively impacted by the increased *T*_water_ and reduced O_2_ levels likely presented by anthropogenic climate change.

## Introduction

Climate change and anthropogenic influences present aquatic organisms with significant challenges (Perry *et al*., 2005; Ficke *et al*., 2007; Diaz and Rosenberg, 2008; Woodward *et al*., 2010). The decline in freshwater quality, as a result of agriculture-driven elevation in nutrient and sediment load, the loss of riparian shade, the presences of continuity barriers (i.e. dams and weirs), combined with increased water temperatures and lowered dissolved O_2_ levels challenge riverine species with multiple stressors. How these challenges affect survival and distribution of economically important fresh water and anadromous fishes, including species native to the British Isles, is of particular interest to conservation physiologists, ecologists and consumers alike (Eaton and Scheller, 1996; Perry *et al.*, 2005; Hari *et al*., 2006; Almodóvar *et al*., 2012). Freshwater fishes are believed to be particularly vulnerable to global fluctuations in atmospheric temperature and its effect on freshwater systems (Eaton and Scheller, 1996; Ficke *et al*., 2007; Woodward *et al*., 2010; Isaak *et al*., 2012). Because of this, the physiological mechanisms underlying the whole animal response to climate change have been studied extensively by fish physiologists, with many recent studies focusing on the effects of increasing environmental temperature on various activities associated with survival (e.g. Portner and Knust, 2007; Clark *et al*., 2008; Farrell *et al*., 2008; Lea *et al.,* 2015).

**Figure 1:**
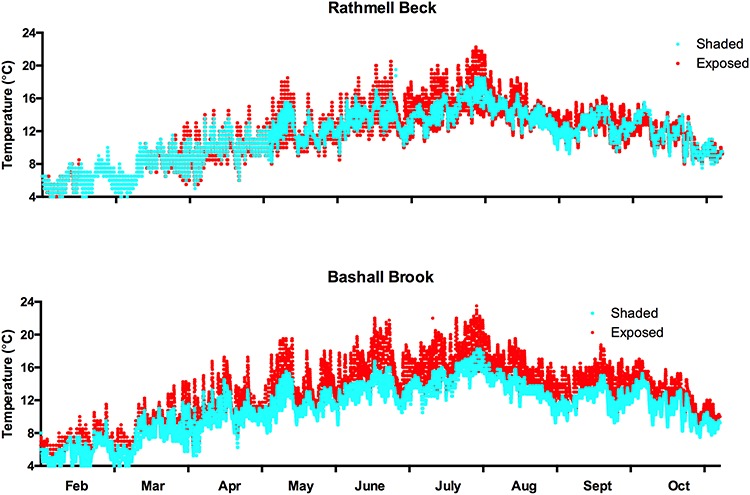
Temperature variation over 9 months in 2014 in two streams in the River Ribble catchment, North West of England. Each data point is a reading from a temperature logger from shaded (blue dots) or exposed (red dots) stretches of Rathmell Beck and Bashall Brook streams. Both streams are known to be inhabited by brown trout, *S. trutta*.

The brown trout, *Salmo trutta*, is an active, cold-water European fish with a distribution ranging from Iceland and arctic Scandinavia to the Mediterranean Sea (Jonsson and Jonsson, 2010). *S. trutta* is an important fisheries and recreational fishing species and as such has been introduced worldwide. Water temperature (*T*_water_ in °C) is believed to be the main abiotic factor limiting the geographical distribution of *S. trutta*, which is commonly found in 5–25°C waters (Elliott and Elliott, 2010). Indeed, *T*_water_ of 4–24°C was recorded in two streams of the Ribble catchment in the UK known to be inhabited by *S. trutta* (Fig. 1). *S. trutta* is also defined as an O_2_ sensitive species, due to its active life history and high O_2_ demands (Jonsson and Jonsson, 2010). Indeed, adequate O_2_ is required to meet the metabolic demands of various life history activities including swimming, feeding, growth, reproduction and predator avoidance. Thus, *S. trutta* is believed to be particularly vulnerable to decreases in environmental O_2_ levels (hypoxia), which is often a direct result of anthropogenic pollution and eutrophication of freshwater habitats, as well as an indirect consequence of decreased O_2_ solubility in water due to increases in *T*_water_ (Landman *et al*., 2005).

Migratory *S. trutta* (sea trout) undertake repeat migrations up and down rivers during spring and autumn where they can encounter changes in water flow, temperature and hypoxia. Thus, understanding how swim speed (*U* in m s^−1^), *T*_water_ and hypoxia affect their swimming efficiency is of utmost importance in order to understand how climate change may affect this species. One way to quantify kinematic efficiency in animals that use oscillatory propulsion (such as tail or wing beats) is to calculate the Strouhal number (St). St is a dimensionless parameter and in fish, and other swimming animals is derived from tail-beat frequency (*f*_tail_), amplitude (*A*) and swimming speed (*U*), which is representative of vortex shedding frequency, wake width and forward velocity, respectively (Nudds *et al*., 2014). Most animals studied to date move within the theoretical optimal range of 0.2 < St < 0.4 (Triantafyllou *et al*., 1991; Triantafyllou *et al.*, 1993), with geometrically similar organisms adhering to similar St (Taylor *et al*., 2003; Nudds *et al*., 2004). Optimal is defined in terms of the highest propulsive efficiency (best mechanical energy input to mechanical energy output ratio). St may decrease slightly with increasing *U* in fish (Hunter and Zweifel, 1971; Webb, 1971; Lauder and Tytell, 2005; Lea et al., 2015), but was also found to increase concomitantly with *U* in rainbow trout, *Oncorhynchus mykiss* (Nudds *et al.*, 2014) and remain constant with *U* in eels, *Anguilla rostrate* (Tytell, 2004). In all cases, however, St appears to remain within the defined optimal range (Nudds *et al*., 2014). Indeed, a change in *T*_water_ was shown to induce changes in *f*_tail_ and *A*, which appeared to be traded off in maintenance of St (Nudds *et al.*, 2014).

Energetic efficiency of locomotion is most often estimated from activity-induced changes in O_2_ consumption (${\dot{M}}_{{\mathsf{O}}_{\mathsf{2}}}$), used as a proxy for whole animal metabolic rate. Efficiency is also measured in relation to the cost of locomotion, often expressed as the cost of transport (COT, in J·km^−1^·kg^−1^). COT provides an index of overall swimming efficiency; the lower the COT value, the more efficient the locomotion (Claireaux *et al*., 2006). In resting animals, increases in *T*_water_ lead to increased ${\dot{M}}_{{\mathsf{O}}_{\mathsf{2}}}$ associated with increased cost of basal metabolic processes (Brett, 1973; Beamish, 1978). Swimming has a similar effect on energy expenditure, with swimming at higher swimming speeds resulting in higher ${\dot{M}}_{{\mathsf{O}}_{\mathsf{2}}}$. The effects of hypoxia on ${\dot{M}}_{{\mathsf{O}}_{\mathsf{2}}}$ are less clear, with fish exhibiting species-specific responses. In salmonids, a decrease in available O_2_ leads to an increase in spontaneous swimming activity and therefore an increase in ${\dot{M}}_{{\mathsf{O}}_{\mathsf{2}}}$, believed to be a behavioural escape response where the fish is seeking higher O_2_ levels (Chapman and McKenzie, 2009).

The independent effects of *T*_water_ or hypoxia on the energetics of swimming performance are well studied in salmonids (Farrell *et al.,* 1998; McKenzie *et al*., 2004; Evans *et al.,* 2007; Clark *et al*., 2011; Norin and Malte, 2012; Svendsen *et al.,* 2012; Eliason *et al.*, 2013), whereas the combined effects of these environmental stressors are rarely examined (Schurmann and Steffensen, 1997; Barnes *et al*., 2011). Even fewer studies have examined the energetics and kinematics of swimming simultaneously (Bushnell *et al*., 1984; Dickson *et al*., 2002; Lea *et al*., 2015). The majority of these studies were conducted under substantial (i.e. <50% air saturation, *P*O_2_ < 10 kPa, [O_2_] < 8 mg/l^−1^ at 10°C and 1 atm) or severe (below critical O_2_ threshold, i.e. <13% air saturation, for rainbow trout, *O. mykiss,*Svendsen *et al.,* 2012) hypoxia. Fewer studies have examined moderate hypoxic conditions (i.e. ~60% air saturation, e.g. Farrell *et al.,* 1998). We know of no studies that have investigated the effect of mild (i.e. ~70% air saturation) hypoxia on salmonid swimming kinematics and metabolism when combined with other environmental stressors relevant to climate change. Because mild hypoxia is increasingly common in un-shaded, slow-flowing parts of rivers where aquatic plants/algae are abundant and because such topology is becoming increasingly common in salmonid waterways due to anthropogenic alteration of physical habitat, such studies are warranted. To our knowledge, this is the first study investigating the combined effects of *T*_water_, mild hypoxia and swimming speed on the energetics and kinematics of swimming in *S. trutta.* It was hypothesized that fish would maintain locomotor mechanical efficiency (St) under combined stressors by modulating their individual kinematic parameters (*f*_tail_ and *A*). Additionally, it was predicted that energetic costs (${\dot{M}}_{{\mathsf{O}}_{\mathsf{2}}}$ and COT) would increase concomitantly with increases in *T*_water_ and decreases in O_2_ saturation.

## Materials and Methods

### Experimental animals

The data presented here are from 18 sub-adult female brown trout (*S. trutta*) with a mean body mass of 236 ± 54 g (range from 172–380 g) and mean body length (BL) of 25.47 ± 1.81 cm (range from 23.0–30.2 cm). Two studies were conducted. The first (study 1) in March of 2014 using 12 fish and the second (study 2) in March of 2015 using 6 fish. In both cases, the fish were collected from Dunsop Trout farm (Dunsop Bridge, Lancashire, England) and kept at the University of Manchester at 10°C (± 1°C) in 500 l recirculating freshwater tanks (8–10 fish per tank) under a daily 12 h:12 h light cycle for at least 4 weeks prior to the start of the experiments. A 10°C acclimation was chosen as it is at the lower end, yet sufficiently above the lower critical range, of the brown trout’s preferred temperature tolerance polygon and allowed for temperature increases of sufficient difference to be tested without approaching the upper incipient lethal temperature (Jonsson and Jonsson, 2010). Fish were fed commercial fish food three times a week. Food, however, was withheld for at least 24 h prior to the start of the experiments so that the trout were post absorptive. All husbandry, housing and experimental protocols were in accordance with UK Home Office legislation for working with animals (project license 40/3584 held by HAS).

### Swimming protocol

A custom built Steffensen-style swim flume (as used in Nudds *et al.*, 2014; Lea *et al*., 2015) was used to measure the energetics and quantify the tail-beat kinematics of swimming trout at different *T*_water_ and speeds and under two different dissolved O_2_ levels. Study 1 used *T*_water_ of 14, 18 and 22°C and study 2 used *T*_water_ of 10, 14 and 18°C. In both cases, dissolved O_2_ levels were 100% and 70% (henceforth high and low) air saturation. *T*_water_ values were selected to provide breadth while remaining within the range experienced by wild trout in the UK during the summer months (Fig. 1). DO recorders were deposited alongside the temperature monitors in the same wild trout streams but these failed. Thus, mild hypoxia at 70% air saturation (*P*O_2 ~_ 14.6 kPa and [O_2_] _~_ 7.91 mg/l^−1^ at 10°C ranging to *P*O_2 ~_ 14.4 kPa and [O_2_] _~_ 6.16 mg/l^−1^ at 22°C) was chosen due to O_2_ dip-probe records from the Ribble River Trust (Gareth Jones, Pers comms) and because this is the level of routine hypoxia commonly found in *S. trutta* in fish farms in the UK (Johansson *et al*., 2006).

The evening before each experiment, a trout was randomly selected, weighed and placed in the swim flume at a water velocity of 0.4 m s^−1^ at 10°C (controlled by a Grant Optima TX150 heating system, Grant Instruments Ltd, Cambridge, UK) and 100% air saturation (normoxia) to acclimatize to the flume. For study 1, the *T*_water_ was increased slowly during the 12-h dark period so that it reached 14°C by the following morning. The following morning, the water velocity was increased from 0.4 m s^−1^ to 0.6 m s^−1^ (low *U*) and the trout swam at this speed for 40 min. Then the velocity was increased to 0.8 m s^−1^ (high *U*) and trout were once more swam for 40 min. These swimming velocities are at the upper range of swimming speeds for salmonids of this size, particularly swimming over this temperature range (Beaumont *et al*., 2000; Altimiras *et al.*, 2002; Lea *et al*., 2015). After this, the speed was turned back down to 0.4 m s^−1^ and the trout rested for 40 min. After this recovery period, a 2-h warming commenced at a rate of 2°C per hour. At 18°C (or 14°C for study 2), the two-speed swimming protocol was repeated, followed by the 40-min recovery and a second warming period up to 22°C (18°C for study 2) over 2 h. The speed was then once more increased to 0.6 and 0.8 m s^−1^. At the end of the 40 min at high *U*, the velocity returned to 0.4 m s^−1^ and the swim flume was rapidly cooled down to 14°C (10°C for study 2). The trout recover at this *T*_water_ overnight while the O_2_ content was slowly changed to 70% air saturation (hypoxia) using an automated gas mixer (International Aqua-Tech Ltd, Gaerwen, Wales, UK) connected to a nitrogen gas cylinder. The following day, the warming/swimming protocol was repeated in the 70% air saturation This time at the end of the warmest *T*_water_ 40 min swimming trial, the trout was removed, placed back in its holding tank and closely monitored, while the swim flume was drained, cleaned, refilled and set ready for the next fish. The order of 70% and 100% saturation days was switched at random to account for any potential training effects. Experimental *T*_water_ was changed in a consistent order. The results obtained, however, were comparable to previous studies (Nudds *et al.*, 2014; Lea *et al*., 2015). Not all fish swam at the highest speed in the highest temperatures—if a fish rested at the back of the working area for an extended period of time, and would not respond to encouragement by a gentle tail poke, the experiment was terminated.

### Kinematics

At all *T*_water_ and at both air saturations, the trout were filmed from above (dorsal view) at 100 frames/second using a Sony HDR-SR8E video recorder (Sony, Tokyo, Japan). Recordings were only taken when the trout were swimming steadily in the selected water velocity (0.6 or 0.8 m s^−1^).

The video footage was imported into Tracker 4.8 video analysis software (Open Source Physics). Clips of three consecutive tail beats were chosen for frame-by-frame digitization of three body parts through time—head, dorsal fin and tail tip. The coordinates of head and tail tip position at each tail beat were used to calculate tail beat amplitude. The midpoint of the tail excursion was taken as a line drawn through the centre of the head parallel to the flow. The max distance that the tail tip reached perpendicularly from this line was then taken as the peak amplitude. Left and right excursions were then summed to give peak-to-peak tail tip amplitude (A). In addition, the time stamp at each video frame was used to calculate tail-beat frequency in Hz (*f*_tail_).

From *A* and *f*_tail_, the St was derived using (Nudds *et al.*, 2014):

 St = *f*_tail_ • A/*U.* (1)

If necessary, adjustments to *U* were made to account for fore–aft positional change between the beginning and end of the video clips.

### Energetics

The mass specific rate of O_2_ consumption (${\dot{M}}_{{\mathsf{O}}_{\mathsf{2}}}$) was used as a proxy for metabolic rate. The energy expenditure for each trout was measured continuously throughout the 2-day experiment using intermittent stop-flow respirometry (Cech and Brauner, 2011; Clark *et al*., 2013). A fibre-optic O_2_ electrode connected to a Witrox 1 O_2_ analyser (Loligo Systems, Viborg, Denmark) was placed in the swim flume to record the O_2_ concentration in the water. Water oxygenation and the flushing pump were controlled through a DAQ-4 data acquisition and relay system (Loligo Systems). Flushing duration was controlled and ${\dot{M}}_{{\mathsf{O}}_{\mathsf{2}}}$ calculated automatically in AutoResp software (Loligo systems) using the following (taken from Clark *et al*., 2013):

${\dot{M}}_{{\mathsf{O}}_{\mathsf{2}}}$ (mg O_2_ kg^−1^ h^−1^) = [(*V*r-*V*_f_) • Δ${C}_{{\mathrm{wo}}_2}$]/(Δ*t* • *M*_f_), (2)

where *V*r is the respirometer volume, *V*_f_ is the fish volume (assuming 1 g of fish is equivalent to 1 ml of water), Δ${C}_{{\mathrm{wo}}_2}$ is change in O_2_ concentration in the closed swim flume, Δ*t* is the time duration (h) and *M*_f_ is trout body mass (kg).

The COT was calculated by converting ${\dot{M}}_{{\mathsf{O}}_{\mathsf{2}}}$ data to J km^−1^ kg^−1^ as described by Claireaux *et al.* (2006) using the oxycalorific coefficient suggested by Beamish (1978) of 1 mg O_2_ = 3.24 Cal and 1 Cal = 4.18 J, and, therefore, 1 mg O_2_ = 13.54 J.

**Figure 2:**
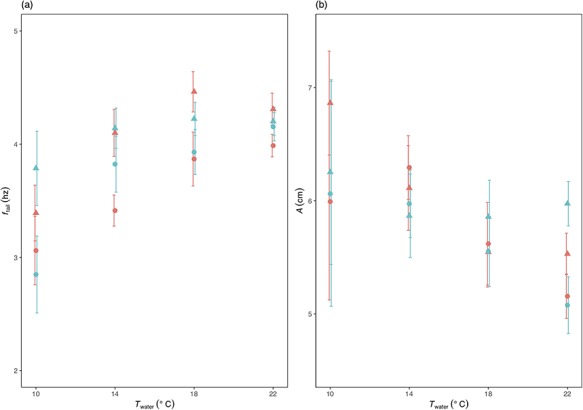
The effect of temperature (*T*_water_) and O_2_ saturation level on (**a**) tail-beat frequency, *f*_tail_ and (**b**) amplitude, *A*, at low and high swimming speeds (*U* = 0.6 and 0.8 ms^−1^) in *S. trutta*. *f*_tail_ was higher at the higher *U* (triangles) than at the low *U* (circles) and increased with increases in *T*_water_. *A* did not differ between the two *U*s, but decreased with increases in *T*_water_. There were no detectable differences between the low O_2_ saturation level (blue symbols) and high O_2_ saturation level (red symbols) on either *f*_tail_ or *A*. The error bars have been shifted across the *x*-axis orientation to improve visual interpretation.

### Data analyses

All data were analysed in R version 3.1.2 (R Core Team, 2014). The data from both studies were pooled together for analyses. Using the lme4 package (Bates *et al.,* 2015), linear mixed effects repeated measures models were generated for each dependent variable (*f*_tail_, *A*, *St*, ${\dot{M}}_{{\mathsf{O}}_{\mathsf{2}}}$ and COT), with *T*_water_, *U* and high O_2_ saturation as fixed factors, and individual trout as a random factor in all cases. All interaction terms were included in the initial models, but were removed if not significant by stepwise backwards deletion. The deletion stopped when either no more interaction terms were non-significant or when only the main effects remained in the statistical model. Interaction terms are only presented in the results when having a significant effect on the dependent variable. All data are plotted as means ± SEM, unless otherwise stated. *P*-values were generated using Type II Wald *X*^2^ tests.

**Figure 3:**
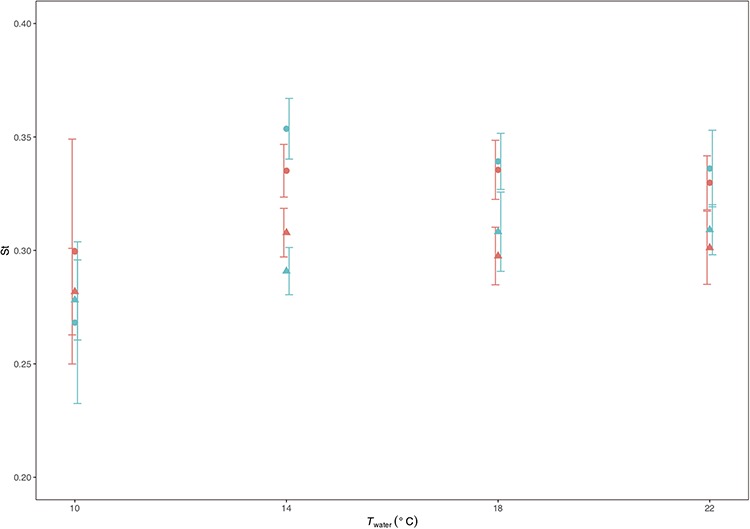
The effects of temperature (*T*_water_) and O_2_ saturation level (low = blue and high = red symbols) on Strouhal number (St) at low (circles) and high (triangles) swimming speeds (*U* = 0.6 and 0.8 ms^−1^) in *S. trutta*. St was lower at the higher *U*, but neither *T*_water_ nor O_2_ saturation level had a detectable effect on St. The error bars have been shifted across the *x*-axis orientation to improve visual interpretation.

The number of trout swum at each temperature were *n* = 6, 18, 18 and 12 at 10, 14, 18 and 22°C, respectively. The *n* is the same for all combinations of speeds and oxygen saturation levels. Final sample sizes, however, differed slightly in the data analyses due to some fish refusing to swim under some treatments. The reluctance to swim did not appear to be size dependent. Although the smallest fish (BL = 23 cm) refused to swim in 22°C at 0.6 m s^−1^ under normoxia and in 22°C at 0.8 m s^−1^ under hypoxia, trout ranging from 25.2 to 25.4 cm also refused to swim under the latter conditions. Furthermore, those trout refusing to swim in other treatment combinations ranged from 25.2 to 26.5 cm BL. The sample sizes for all kinematic parameters (Figs 2 and 3) for normoxia are *n* = 4, 18, 18 and 11 at 10, 14, 18 and 22°C for low *U* (0.6 m s^−1^) swimming and *n* = 6, 18, 17 and 11 at 10, 14, 18 and 22°C, respectively, for high *U* (0.8 m s^−1^) swimming. For hypoxia, they are *n* = 6, 18, 17 and 12 at 10, 14, 18 and 22°C for low *U* swimming and *n* = 6, 17, 17, 9 at 10, 14, 18 and 22°C, respectively, for high *U* swimming.

**Figure 4:**
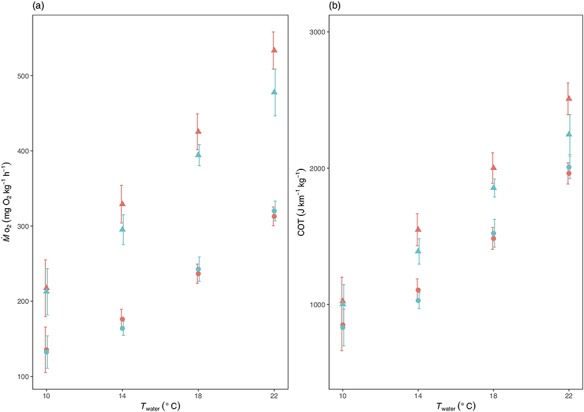
The effect of temperature (*T*_water_) and O_2_ saturation level on (**a**) O_2_ consumption, ${\dot{M}}_{{\mathsf{O}}_{\mathsf{2}}}$, and (**b**) cost of transport, COT, at low and high swimming speeds (*U* = 0.6 and 0.8 m s^−1^) in *S. trutta*. ${\dot{M}}_{{\mathsf{O}}_{\mathsf{2}}}$ was higher at the higher *U* (triangles) than at the lower *U* (circles). ${\dot{M}}_{{\mathsf{O}}_{\mathsf{2}}}$ also increased with increases in *T*_water_. The only detectable difference in ${\dot{M}}_{{\mathsf{O}}_{\mathsf{2}}}$between O_2_ saturation levels was at the highest *U* and *T*_water_ combination, where ${\dot{M}}_{{\mathsf{O}}_{\mathsf{2}}}$ was found to be lower in the low O_2_ saturation level (blue) than in the higher O_2_ saturation level (red) conditions. The results for COT are similar to those seen for ${\dot{M}}_{{\mathsf{O}}_{\mathsf{2}}}$. The error bars have been shifted across the *x*-axis orientation to improve visual interpretation.

For both ${\dot{M}}_{{\mathsf{O}}_{\mathsf{2}}}$ and COT (Fig. 4) in normoxia sample sizes were *n* = 6, 18, 18 and 12 at 10, 14, 18 and 22°C for low *U* (0.6 m s^−1^) swimming and *n* = 6, 18, 17 and 12 at 10, 14, 18 and 22°C, respectively, for high *U* (0.8 m s^−1^) swimming. In hypoxia, *n* = 6, 18, 17 and 11 at 10, 14, 18 and 22°C for low *U* swimming and *n* = 6, 17, 17 and 11 at 10, 14, 18 and 22°C, respectively, for high *U* swimming.

## Results

### Kinematics

*f*_tail_ (Fig. 2a and Table 1) was greater at high *U* (*X*^2^ = 24.256, *P* < 0.001) and increased with increasing *T*_water_ (*X*^2^ = 5.237, *P* = 0.022). In contrast, O_2_ saturation level had no detectable effect on *f*_tail_ (*X*^2^ = 1.006, *P* = 0.316). *A* (Fig. 2b and Table 1) was not affected by either *U* (*X*^2^ = 0.898, *P* = 0.343) or O_2_ level (*X*^2^ = 0.304, *P* = 0.581), but decreased with increasing *T*_water_ (*X*^2^ = 3.946, *P* = 0.047). There was no detectable effect of either *T*_water_ (*X*^2^ = 1.570, *P* = 0.210) or O_2_ saturation level (*X*^2^ = 0.077, *P* = 0.782) on St. St, however, was lower at the higher *U* (*X*^2^ = 20.834, *P* < 0.001).

**Table 1:** Means ± standard error for each of the dependent variables and each combination of independent variable (treatments)

Independent variables			Dependent variables				
*T* (°C)	Oxygen saturation (%)	*U* (m s ^−1^)	${\dot{M}}_{{\mathsf{O}}_{\mathsf{2}}}$ (mg O2 kg^−1^ h^−1^)	COT	*f*_tail_ (Hz)	*A* (cm)	St
10	70	0.6	132.35 ± 21.51	829.81 ± 134.86	2.85 ± 0.34	6.06 ± 0.99	0.27 ± 0.04
		0.8	212.76 ± 30.85	1000.52 ± 145.07	3.79 ± 0.33	6.25 ± 0.82	0.28 ± 0.02
	100	0.6	135.42 ± 30.15	849.08 ± 189.04	3.06 ± 0.30	5.99 ± 0.87	0.30 ± 0.05
		0.8	217.37 ± 37.64	1022.17 ± 176.98	3.39 ± 0.25	6.86 ± 0.46	0.28 ± 0.02
14	70	0.6	163.91 ± 9.40	1027.72 ± 58.94	3.82 ± 0.25	5.97 ± 0.30	0.35 ± 0.01
		0.8	295.23 ± 19.80	1388.30 ± 93.09	4.14 ± 0.18	5.87 ± 0.37	0.29 ± 0.01
	100	0.6	176.22 ± 13.07	1104.90 ± 81.97	3.41 ± 0.14	6.29 ± 0.28	0.34 ± 0.01
		0.8	329.04 ± 25.01	1547.30 ± 117.62	4.10 ± 0.21	6.11 ± 0.37	0.31 ± 0.01
18	70	0.6	242.66 ± 16.24	1521.46 ± 101.83	3.93 ± 0.20	5.55 ± 0.31	0.34 ± 0.01
		0.8	394.30 ± 14.08	1854.19 ± 66.23	4.22 ± 0.15	5.86 ± 0.32	0.31 ± 0.02
	100	0.6	236.60 ± 12.82	1483.46 ± 80.36	3.87 ± 0.24	5.62 ± 0.36	0.34 ± 0.01
		0.8	425.40 ± 23.87	2000.45 ± 112.27	4.46 ± 0.18	5.55 ± 0.31	0.30 ± 0.01
22	70	0.6	320.07 ± 13.23	2006.86 ± 82.93	4.15 ± 0.12	5.08 ± 0.25	0.34 ± 0.02
		0.8	477.71 ± 31.16	2246.44 ± 146.52	4.20 ± 0.12	5.97 ± 0.20	0.31 ± 0.01
	100	0.6	312.85 ± 12.50	1961.60 ± 78.39	3.99 ± 0.10	5.16 ± 0.20	0.33 ± 0.01
		0.8	533.48 ± 24.76	2508.71 ± 116.44	4.31 ± 0.14	5.53 ± 0.18	0.30 ± 0.02

### Energetics

${\dot{M}}_{{\mathsf{O}}_{\mathsf{2}}}$ (Fig. 4a and Table 1) was greater at the higher swimming speed than at the lower speed (*X*^2^ = 349.239, *P* < 0.001) and increased with increases in *T*_water_ (*X*^2^ = 267.628, *P* < 0.001). Taken together across all temperatures, ${\dot{M}}_{{\mathsf{O}}_{\mathsf{2}}}$ was similar in both oxygen saturation levels (*X*^2^ = 3.122, *P* = 0.077). There was, however, a higher incremental increase (interaction between temperature and *U*) in ${\dot{M}}_{{\mathsf{O}}_{\mathsf{2}}}$ with *T*_water_ at the higher speed (*X*^2^ = 10.679, *P* < 0.001). A significant interaction between *U* and O_2_ saturation level (*X*^2^ = 3.878, *P* = 0.049) indicated that the effect of mild hypoxia on ${\dot{M}}_{{\mathsf{O}}_{\mathsf{2}}}$ was inconsistent between speeds with low saturation inducing a lower measured ${\dot{M}}_{{\mathsf{O}}_{\mathsf{2}}}$ at the higher speed, whereas oxygen saturation level did not detectably affect ${\dot{M}}_{{\mathsf{O}}_{\mathsf{2}}}$ at the lower speed.

COT (Fig. 4b and Table 1) was higher at the higher swimming speed than at the lower speed (*X*^2^ = 82.946, *P* < 0.001) and increased with increases in *T*_water_ (*X*^2^ = 289.100, *P* < 0.001), but it was not affected by oxygen saturation level (*X*^2^ = 2.494, *P* = 0.114).

## Discussion

Swimming is an energetically costly behaviour and a fundamental part of *S. trutta* life history. The aim of this study was to determine the combined effects of *T*_water_, O_2_ saturation level and swimming speed on the tail-beat kinematics and energetics of brown trout, with applicability to climate change. Arguably, two of the findings are particularly pertinent to this aspect; both ${\dot{M}}_{{\mathsf{O}}_{\mathsf{2}}}$ and St give an indication of whether the trout are able to tolerate the range of conditions experienced in terms of aerobic respiration for the former and propulsive efficiency for the latter. We found that St was not affected by either changes to *T*_water_ or O_2_ saturation level that is consistent with the growing body of work showing that fish maintain St in an optimal (or preferred) range (Hunter and Zweifel, 1971; Webb, 1971; Taylor *et al*., 2003; Lauder and Tytell, 2005; Nudds *et al.*, 2014; Lea *et al*., 2015) (Fig. 3). Thus, it appears that brown trout can maintain propulsive efficiency throughout the natural *T*_water_ and O_2_ saturation ranges currently experienced. Similarly, for the most part, neither current *T*_water_ nor O_2_ saturation levels are likely to present an energetic obstacle. This is with the exception of the highest *T*_water_ and low O_2_ saturation combination, where ${\dot{M}}_{{\mathsf{O}}_{\mathsf{2}}}$ was found to drop (Fig. 4a) (discussed further below).

Strouhal number (St) was marginally lower at the high *U*, similar to findings from previous studies on fish (Hunter and Zweifel, 1971; Webb, 1971; Lauder and Tytell, 2005; Lea *et al*., 2015), with the trout maintaining St within the theoretical range for mechanical efficiency of 0.2 to 0.4 (Triantafyllou *et al*., 1991; Triantafyllou *et al.*, 1993; Taylor *et al*., 2003) in all combinations of *T*_water_, O_2_ saturation level and *U*. The data indicate no effect of O_2_ saturation level on swimming kinematics in the trout, but *f*_tail_ increased and *A* decreased with increasing *T*_water_. Here, and as in previous studies, a *T*_water_ driven trade-off between *f*_tail_ and *A* appears to be initiated in order to maintain a preferred St. The exact nature of this trade-off, however, is not necessarily consistent among studies. Increases in *T*_water_ may lead to concomitant increases in *f*_tail_ as found this present study (Stevens, 1979; Nudds *et al.*, 2014; Lea *et al*., 2015), decreases (Stevens, 1979) in *f*_tail_ or, alternatively, no detectable effect (Rome *et al*., 1990). Similarly, as found here, increases in *T*_water_ were previously linked to decreases in *A* for salmonids (Nudds *et al.*, 2014; Lea *et al*., 2015), yet had no discernable effect on *A* in carp (Rome *et al*., 1990). Determining whether these differences are due to methodology or taxa requires further work.

Swimming represents a considerable component of the energy budget of active fishes, and high intensity aerobic swimming can utilize a significant proportion of aerobic metabolic scope (Claireaux and Lefrançois, 2007; Chapman and McKenzie, 2009). As predicted, ${\dot{M}}_{{\mathsf{O}}_{\mathsf{2}}}$ increased with both swimming speed and temperature, but O_2_ saturation level had no detectable effect. The ${\dot{M}}_{{\mathsf{O}}_{\mathsf{2}}}$ values presented in this study are well within the range of those presented in other salmonid studies carried out at similar temperatures (e.g. Beaumont *et al*., 2000; Altimiras *et al.*, 2002; Lee *et al.*, 2003; Clark *et al*., 2011; Eliason *et al*., 2013). Calculating COT without accurate CO_2_ production measurements has its limitations. Nevertheless, the values calculated here are within the range of values seen in other athletic fish species at similar *U* and at similar *T*_water_, such as the European sea bass, *Dicentrarchus labrax* (Claireaux *et al*., 2006).

The lack of a detectable change in ${\dot{M}}_{{\mathsf{O}}_{\mathsf{2}}}$ with O_2_ saturation level at all but the highest *T*_water_, *U* and lowest O_2_ saturation level deserves further consideration. It is possible that 70% air saturation is not hypoxic enough to induce a change in swimming physiology in the brown trout at anything other than the more extreme conditions their wild counterparts encounter. Under these conditions, it is possible the fish switched from aerobic to anaerobic metabolism, with overall metabolism increasing despite the observed reduction in O_2_ uptake. Indeed, previous studies suggest fish may start switching to anaerobic metabolism when swimming at just 70% of their *U*_crit_ (Burgetz *et al*., 1998). It may be that in the most extreme combination of the conditions used here, the fish were approaching the limit for sustainable steady-state swimming. Indeed, our highest *U* was just below burst and glide locomotion. During hypoxia and intense swimming, salmonid glycolytic white muscle produces significant amounts of lactate, more than can be processed by aerobic tissues like red muscle and the heart (Weber *et al.,* 2016). Additionally, the fish may have reduced blood flow to organs like the liver and the gut during the more extreme swimming conditions that may have reduced over all O_2_ consumption (Thorarensen and Farrell, 2006). Whether, however, these processes account for the lowered ${\dot{M}}_{{\mathsf{O}}_{\mathsf{2}}}$ observed here, or had an effect, albeit lesser, at the less extreme combinations of the treatments is not known. Previous studies show that mild hypoxia can have a significant negative effect on repeated swimming performance in mature wild caught sockeye salmon, *Oncorhynchus nerka* (Farrell *et al*., 1998). Therefore, it may be that hypoxia can be accommodated by the fish over the duration of a single swimming event as used here, whereas over multiple swims and longer swim durations, changes in O_2_ saturation may have detrimental effects. It should, however, also be noted that swimming performance is not the only trait that may be limited by hypoxia. In Atlantic salmon (*Salmo salar*) aquaculture, fish exposed to cyclic episodes of hypoxia at O_2_ concentrations of 6 mg/l and lower (air saturation equivalent of 66–78% DO at *T*_water_ of 10–18°C) show signs of physiological changes such as sub-optimal growth (Farrell and Richards, 2009; Burt *et al*., 2012).

Multiple statistical tests were conducted within this present study and two *P*-values that are only just below the *α* = 0.05 level are interpreted as significant effects. We would argue that the relatively small sample sizes, would mean any correction for a false discovery rate (Curran-Everett, 2000) would be too conservative. Furthermore, one of these *P*-values (*P* = 0.047), relates to a decrease in *A* with *T*_water_, which is supported logically. Specifically, because St remained unaffected by, whilst *f*_tail_ increased with increases in *T*_water_, clearly *A* had to decrease with increasing *T*_water_, because St is a product of *f*_tail_ and *A* (when *U* is fixed). Hence, we would argue that the conclusion of a significant effect here is robust. The second *P*-value (*P* = 0.049) relates to the interaction between *U* and O_2_ saturation level on ${\dot{M}}_{{\mathsf{O}}_{\mathsf{2}}}$, which is interpreted as an indication that the trout were perhaps switching from aerobic to anaerobic metabolism at the highest *T*_water_ and *U* combination in the low O_2_ saturation level condition. Although this result does not have the logic-based support of the *A* and *T*_water_ result, it, nonetheless, is certainly not an unexpected finding.

Both warming waters and hypoxia are O_2_-limiting stressors for energetically demanding activities such as locomotion (Claireaux and Lefrançois, 2007; Ejbye-Ernst *et al.*, 2016; Ern *et al.*, 2016). To our knowledge this study is the first to examine tail-beat kinematics and the energetics of swimming under combined environmental stressors in a species of fish. Kinematic (mechanical) efficiency was not compromised, with no change in St detected in response to *T*_water_ changes or O_2_ saturation level. However, the energetic cost of locomotion (${\dot{M}}_{{\mathsf{O}}_{\mathsf{2}}}$), increased with increases in *T*_water_ and with higher *U*. Mild hypoxia had no detectable effect on swimming performance except at the most extreme combination of conditions experienced. Future studies should expand the range of the conditions tested in this study to further probe the vulnerability of this ionic fish. Indeed, our data suggests that if anthropogenic climate change pushes conditions beyond those used in this study and the trout are unable to either migrate or avoid compromised areas, they will experience detrimental levels of physiological stress.
